# To mate or not to mate

**DOI:** 10.7554/eLife.13093

**Published:** 2015-12-23

**Authors:** Florencia Campetella, Silke Sachse

**Affiliations:** Department of Evolutionary Neuroethology, Max Planck Institute for Chemical Ecology, Jena, Germanyfcampetella@ice.mpg.de; Department of Evolutionary Neuroethology, Max Planck Institute for Chemical Ecology, Jena, Germanyssachse@ice.mpg.de

**Keywords:** pheromone, neural circuit, sensory processing, mating behavior, *D. melanogaster*

## Abstract

Mechanisms of the neural circuits that guide mating decisions in male flies are becoming clearer.

**Related research article** Kallman BR, Kim H, Scott K. 2015. Excitation and inhibition onto central courtship neurons biases *Drosophila* mate choice. *eLife* **4**:e11188. doi: 10.7554/eLife.11188

**Image** Pathways in a male fly’s nervous system process information about pheromones

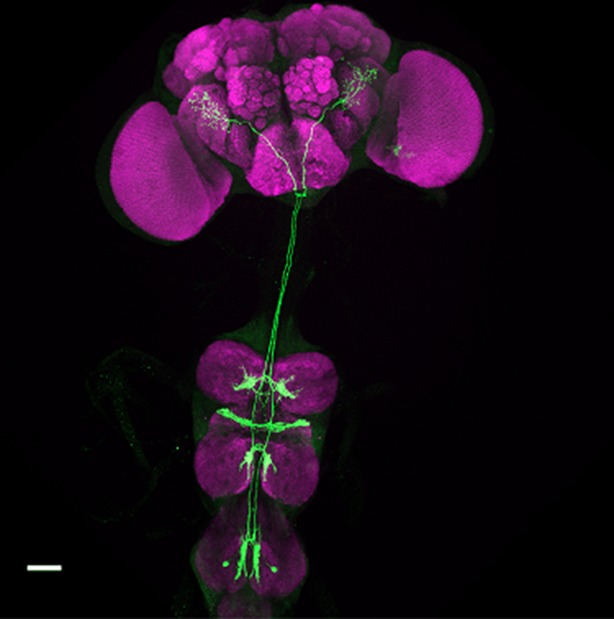

The theory of natural selection is often summarised with the phrase ‘the survival of the fittest’. But simply surviving is not enough; organisms must also reproduce, otherwise there would be no evolution. In the animal kingdom, for example, colourful feathers, sophisticated songs and elaborate courtship rituals are all employed to increase the chances of a successful copulation. Naturalists have marvelled at these mating rituals for hundreds of years and filled countless notebooks with descriptions and drawings of them. However, a more detailed understanding of mating behaviour has had to wait for arrival of new techniques in genetics and neuroscience, and the rise to fame of the vinegar fly, *Drosophila melanogaster*, as a model organism.

Vinegar flies (or common fruit flies) perform an elaborate courting ritual: first, a male orients towards and follows a female; then he touches her abdomen with his foreleg and, if she responds, he extends one of his wings and performs a song-like vibration with it. Finally he licks the female’s genitalia and attempts to copulate with her. Now, in eLife, Benjamin Kallman, Heesoo Kim and Kristin Scott of the University of California at Berkeley identify further pathways that guide mating decisions in male flies (Kallman et al., 2015).

With the discovery of the transcription factor Fruitless (which is expressed only in males, and called FruM for short), we now know that around 1,500 neurons orchestrate courtship behaviour in these flies (Manoli et al., 2005; Stockinger et al., 2005). However, little is known about how these neurons are connected to achieve this behaviour. Previous studies have shown that chemicals present on the cuticle can affect courtship behaviour in flies (Yamamoto and Koganezawa, 2013). The cuticular pheromones produced by a female fly promote mating and allow the courting male to identify her as a female of the same species; the pheromones produced by males have the opposite effect and inhibit courtship. Flies detect these compounds via a pair of sensory neurons housed in their leg bristles. The neurons that respond to male pheromones are called M cells, and those that respond to female pheromones are called F cells (Figure 1; Pikielny, 2012; Thistle et al., 2012).

**Figure 1.**
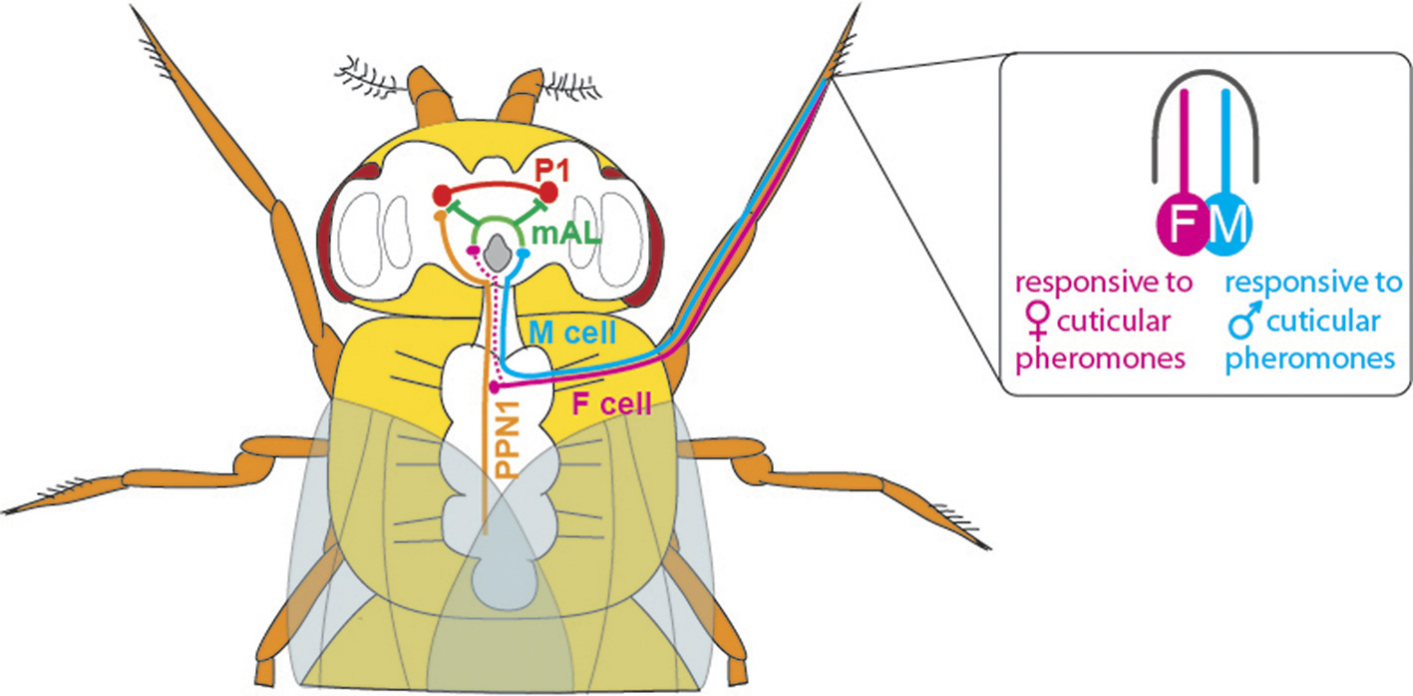
The neuronal circuitry underlying male-specific courtship behaviour in flies. The leg bristles of flies contain neurons called F cells (shown in pink) that respond to female pheromones, and M cells (blue) that respond to male pheromones. Kallman et al. show that the F cells connect with PPN1 neurons (orange) in the ventral nerve cord; the PPN1 neurons then activate P1 neurons (red) in a part of the higher brain called the protocerebrum. The M cells project to the mAL neurons (green), which inhibit the P1 neurons. The F cells also indirectly activate the mAL neurons (represented by the dotted line).

Kallman et al. used genetic and behavioural tools to show that activating the F and M cells can modify male courtship behaviour. In a second step, they were able to identify the connection between the F and M cells in the leg and a cluster of neurons (called P1 neurons) that is found only in the brain of male flies. Neurobiologists have become increasingly interested in P1 neurons over the last decade, and have shown that activating them correlates with the start of courtship behaviour (Clowney et al., 2015; Kohatsu et al., 2011). However, until recently, the roads that lead to P1 were hardly known.

Kallman et al. then monitored activity in the P1 cluster when they activated either F cells or M cells in the forelegs of male flies. F cell activation was seen to excite the P1 cluster and resulted in enhanced courtship, whereas M cell activation did not excite the P1 cluster and may even have suppressed it. However, given that F cells terminate in the fly’s thorax, Kallman et al. found that another class of neurons, called PPN1, serves as the link between F cells and the P1 cluster in the higher brain (Figure 1).

To uncover the targets of the M cells, Kallman et al. looked at the neurons that express the FruM transcription factor and observed how their activity changed when M cells were stimulated. They found that the M cells activated a cluster of neurons called mAL (Figure 1); this cluster had previously been shown to send out inhibitory signals (Kimura et al., 2005). Inactivating the mAL neurons led to males courting each other. But how does mAL normally inhibit male-male courtship? In a final set of experiments, Kallman et al. showed that mAL neurons form connections with P1 neurons and inhibit their activity. Thus, while activating F cells led to an overall activation of P1, activating M cells resulted in an overall inhibition of P1. This suggests that the balance between excitation and inhibition determines this male-specific behaviour.

The work of Kallman, Kim and Scott represents a beautiful example of how sensory information can be processed by a very restricted number of neurons to generate highly complex forms of behaviour. The future will tell whether this is a general feature of sensory networks.

## References

[bib1] Clowney EJ, Iguchi S, Bussell JJ, Scheer E, Ruta V (2015). Multimodal chemosensory circuits controlling male courtship in *Drosophila*. Neuron.

[bib2] Kallman BR, Kim H, Scott K (2015). Excitation and inhibition onto central courtship neurons biases *Drosophila* mate choice. eLife.

[bib3] Kimura K-I, Ote M, Tazawa T, Yamamoto D (2005). Fruitless specifies sexually dimorphic neural circuitry in the *Drosophila* brain. Nature.

[bib4] Kohatsu S, Koganezawa M, Yamamoto D (2011). Female contact activates male-specific interneurons that trigger stereotypic courtship behavior in *Drosophila*. Neuron.

[bib5] Manoli DS, Foss M, Villella A, Taylor BJ, Hall JC, Baker BS (2005). Male-specific fruitless specifies the neural substrates of *Drosophila* courtship behaviour. Nature.

[bib6] Pikielny CW (2012). Sexy DEG/ENaC channels involved in gustatory detection of fruit fly pheromones. Science Signaling.

[bib7] Stockinger P, Kvitsiani D, Rotkopf S, Tirián L, Dickson BJ (2005). Neural circuitry that governs *Drosophila* male courtship behavior. Cell.

[bib8] Thistle R, Cameron P, Ghorayshi A, Dennison L, Scott K (2012). Contact chemoreceptors mediate male-male repulsion and male-female attraction during *Drosophila* courtship. Cell.

[bib9] Yamamoto D, Koganezawa M (2013). Genes and circuits of courtship behaviour in *Drosophila* males. Nature Reviews Neuroscience.

